# Reconfigurable, Defect-Free, Ultrahigh-*Q* Photonic Crystal Microcavities for Sensing

**DOI:** 10.3390/s130303262

**Published:** 2013-03-08

**Authors:** Snjezana Tomljenovic-Hanic, C. Martijn de Sterke

**Affiliations:** 1 School of Physics, University of Melbourne, Parkville, VIC 3010, Australia; 2 ARC Centre of Excellence for Ultrahigh-bandwidth Devices for Optical Systems, and School of Physics, University of Sydney, Sydney, NSW 2006, Australia; E-Mail: desterke@physics.usyd.edu.au

**Keywords:** photonic crystal slab, microcavity, infiltration, sensing

## Abstract

We propose a new approach for creating reconfigurable high-*Q* cavities in defect-free photonic crystal slabs (PCSs). The approach relies on selective air-hole infiltration in otherwise defect-free PCSs. We show that using this method we can design ultrahigh-*Q* microcavities, with *Q*˜10^6^. Numerical calculations indicate a large number of high-*Q* modes with high sensitivity, which are ideal for simultaneous, multi-parameter refractive index-based sensing.

## 1. Introduction

Since 2004, photonic crystal cavities have been extensively studied. A large improvement in performance, as measured by the increased quality factors and small modal volumes has been achieved. The highest quality factors were obtained with double-heterostructure type cavities [1–10]. In their original form, these cavities are formed by shifting the air-holes in the central part across the waveguide. However, this type of cavity requires high-precision and offers little prospect for post-processing. Other methods, in which cavities are formed by air-hole infiltration [3,6], changes of the refractive index of the dielectric (chalcogenide glass [5,7] or silicon [10], do not rely on high precision fabrication [3–5] and have been experimentally demonstrated [6–10]. In related work, a photonic crystal cavity was produced in an otherwise defect-free photonic crystal slab by using a relocatable fibre taper [9], by changing the refractive index of a slab [10], or by presence of a nanoparticle [11]. Here we extend this approach to forming high-*Q* cavities in an otherwise defect-free PCS by selective infiltration of the air-holes. Such cavities can easily be modified and reconfigured. This approach places no additional limitations on the materials used either for the PCS fabrication or for the infiltration. Modification of PCS cavities by infiltration has been used extensively with a variety of materials used for infiltration such as water [12], polymer [13,14] and liquid crystal [15–17]. While we mainly consider infiltration by water, we make some comparisons with cavities formed by polymer infiltration. Though it is challenging to infiltrate air-holes with diameters below 300 nm, a method to achieve this has been reported, showing infiltration even of a single-hole [18]. The precision of this process is mainly limited by the amount of fluid residing on the microtip which is used for the infiltration.

## 2. Model and Method

**Figure 1.**
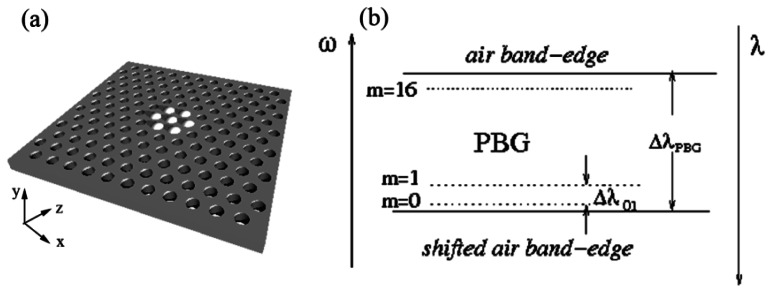
Schematic of (**a**) a defect-free PCS with infiltrated holes indicated in white; (**b**) The mode gap, the gap between the original and the shifted air band-edge, with the frequency of the localized modes superimposed.

A schematic of the geometry is shown in Figure 1(a). We consider a two-dimensional photonic crystal slab with a hexagonal array of cylindrical air-holes in silicon slab with *n* = 3.4. The period is *a*, the radius of the holes is *R* = 0.29*a*, and the thickness of the slab is *h* = 0.6 *a*. The structure has 51 periods both in the *x*- and the *y*-directions. The infiltrated region is centered at the central hole of the slab. A larger cavity is obtained by infiltrating the 6 rings of holes surrounding this central hole (see Figure 1(a)). Additional rings of holes can be added in a similar way. We assume the holes to be filled completely, without meniscus. For most results presented here the number of rings that are infiltrated around the central hole is six and water (*n_h_* = 1.33) is used for infiltration. We then briefly compare these results with those for cavities formed using smaller numbers of infiltrated rings or using different material for infiltration (polymer with refractive index *n*_h_ = 1.45).

Infiltrating the air-holes with a liquid increases the average refractive index in a finite infiltrated region. By the process first demonstrated in [19], when the refractive index is increased, the bands of PCS shift to lower frequencies. The mode forming at the top of the band gap (the air band-edge mode) enters the photonic band gap (PBG) of the unaffected structure surrounding this region, see Figure 1(b). This results in a mode being localized to the region with the change in refractive index—this region thus forms a cavity. In the mode gap between the original frequency and shifted air band edge frequency a large number of localized modes can be induced, in our case as many as 16, as illustrated in Figure 1(b). The number of localized modes increases with the refractive index difference between air and the infiltrating liquid and with the cavity's area.

The three-dimensional Plane Wave Expansion Method is used for photonic band gap calculations and the three-dimensional Finite-Difference Time-Domain on a cluster of machines is used for the cavity resonances calculations. The computational window is reduced eight times using the symmetry properties of the field. As the field is symmetric in the vertical direction there are four possible symmetries to be considered: SSS, SSA, ASS and ASA, with symmetries denoted in the (*x*,*y*,*z*) direction, where S stands for symmetric and A stands for anti-symmetric. Satisfactory convergence is obtained by using 34 points per period for ultrahigh-*Q* modes (*Q* > 10^6^) and 32 points per period for high-*Q* modes (*Q* < 10^6^). The perfectly-matched layer width and the height of the computational window are chosen to be 2*a* and 4*h*, respectively. The modal volume and partial quality factors are obtained by post-processing. The modal volume is calculated using:

(1) ∭UdV/max(U)

where U = ɛ |E| ^2^/2 is the electric energy density.

## 3. Results and Discussion

In Table 1 we summarize results for quality factors, symmetries, frequencies, and modal volumes of the 16 localized high-*Q* modes induced by filling six rings of holes around a central hole with water, in total 127 infiltrated holes.

Note that all four mode symmetries are equally represented and that for almost all modes *Q* > 1 × 10^6^. For all four possible symmetries there are high and ultrahigh-*Q* modes and all of them are limited by out-of-plane losses. For example, the in-plane *Q* for the SSA_0_ mode is *Q_II_* = 10^8^ and the limiting out-of-plane component is *Q_⊥_* = 1.01 × 10^6^. This ratio of the partial *Qs* contribution is similar for all modes presented here. The highest quality factors are obtained for the second set of modes (with subscript 1 in Table 1), for which *Q* > 2 × 10^6^ for all four symmetries. The modal volume is in the range 1.68 to 4.89 (*λ*/*n*)^3^. These values are considered relatively small for the band-edge type modes. The modes' resonant wavelengths are calculated for a fixed period *a* = 480 nm in order to operate around *λ* = 1,550 nm. For this particular periodicity mode spacing ranges between 0.4 nm and 10.6 nm.

**Figure 2.**
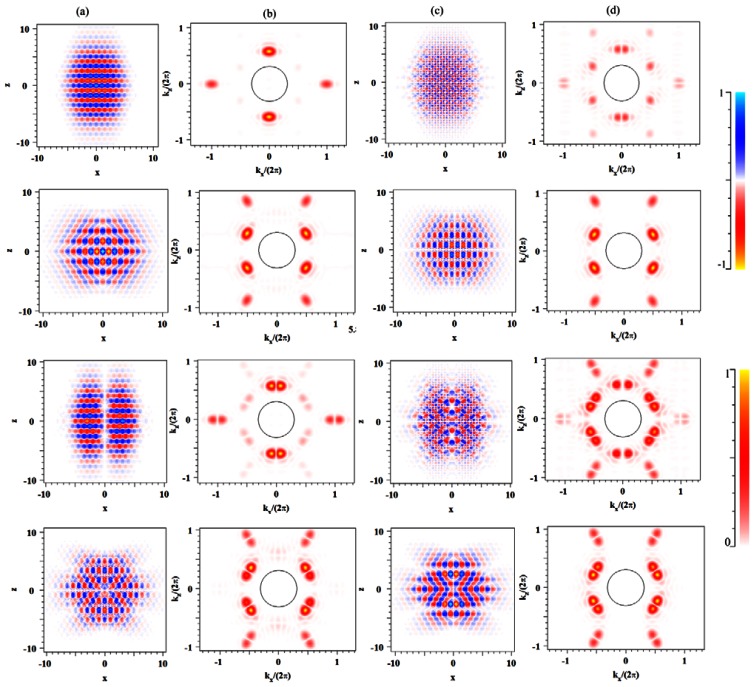
The major electric field components, (**a**) *E_x_* and (**c**) *E_z_*, in the plane and its Fourier transforms (**b**) *E_x_* and (**d**) *E_z_* for the first four ultrahigh-*Q* cavity modes SSS_0_, ASA_0_, ASS_0_ and SSA_0_. The number of infiltrated circles around the central hole is *N* = 6 and the refractive index of the infiltrated holes is *n_h_* = 1.33.

In Figure 2(a,c) we show the major electric field components *E_x_* and *E_z_*, in the plane for the first four ultrahigh-*Q* cavity modes, namely SSS_0_, ASA_0_, ASS_0_ and SSA_0_. Though not shown on the scale of the figures, a large fraction of the field is located within air-holes as expected for the air-band edge modes. In Figure 2(b,d) we show the Fourier transforms of Figure 2(a,c), respectively, with a circle indicating the light-cone. The field within the leaky light-cone region is insignificant as required for high-*Q* cavity modes [20].

So far we have shown results for cavities with six rings infiltrated around the central hole. Decreasing the cavity size decreases the *Q*. For example varying the number of rings around the central hole infiltrated from two to six the quality factor increases from *Q* = 5.50 × 10^5^ to *Q* = 1.20 × 10^6^ for the ASA_1_ mode. Decreasing the size of the infiltrated region decreases the *Q*, but the modal volume does not change significantly. This behaviour is similar to that of infiltrated double-heterostructure type cavities [3]. Increasing the refractive index of infiltrated material decreases the *Q*. For example, if the cavity is formed by material of the refractive index of *n_h_* = 1.45, corresponding to a typical polymer, the *Q* of the ASA_1_ mode decreases from *Q* = 1.20 × 10^6^ to *Q* = 6.29 × 10^5^. This decrease is due to out-of-plane losses, as the refractive index difference between the air-silicon and liquid-silicon is decreased.

We have found that the cavity properties seem to be tolerant to variations in the hole filling, even if a few holes in the cavity region are not infiltrated. Similar findings were experimentally verified for the infiltrated double-heterostructure type cavities [6]. In practice, both the slab and the liquid can be absorbing which may affect the *Q* of the cavity [21,22]. For example, water has high absorption losses at wavelengths above 1 μm. Therefore the fluid should be carefully chosen with regards to the wavelengths of operation [21]. For future experimental verification, it is important to realize that the properties of both the surface of the slab and the fluid, determine the penetration of the fluid in the holes.

### Application to Sensing

Compared to dielectric band edge modes, air-band edge modes are more sensitive to variations in the refractive index of the holes since the field is concentrated there, but they usually have smaller *Q* [21,22]. Therefore there is a trade-off for refractive index-based sensing [21,22]. Recently, we reported ultrahigh-*Q* air-band edge cavities formed by the induced refractive index change in chalcogenide-based PCS [5]. However, when these cavities are infiltrated their *Q* significantly degrades. Quite the opposite happens with the cavity formed by infiltration which we consider here: instead of degrading the cavity the infiltration induces the ultrahigh-*Q* cavity.

We investigate the sensitivity of this type of cavity considering a small refractive index change Δ*n_s_* induced by the presence of the sample in the water. The sensitivity *S* is calculated using method described in Reference [21]. The sensitivity of the mode is defined as the ratio of the frequency shift *Δω* and the spectral linewidth *δω*:

(2) $$S=\frac{\Delta \omega }{\delta \omega }.$$

where δω = ω/Q.

**Figure 3.**
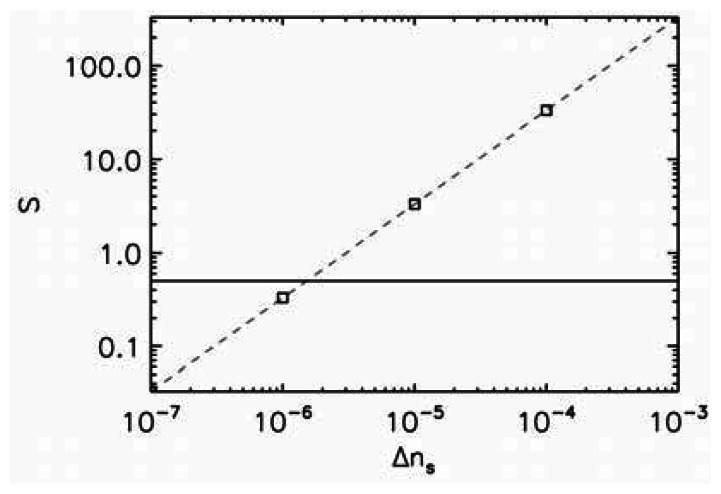
Sensitivity as a function of the refractive index change induced by a presence of the sample for all of the ultrahigh-*Q* modes SSS_1_, ASA_1_, ASS_1_, and SSA_1_ in Table 1. The shift for all these modes is essentially the same. The horizontal line represents the detection limit; above the line the refractive index change induced by the sample is detectable.

In Figure 3 we plot the sensitivity as a function of the refractive index change due to the presence of the sample. The horizontal line corresponds to the detection limit of *S* = 0.5. Therefore, everything above that line is detectable. We choose the second set of modes (*i.e.*, the modes with subscript 1) as they have the highest quality factors. The detection limit for these modes is essentially the same so only one set of data is shown; for these modes the sensitivity is close to 10^−6^. The fact that these modes have the same sensitivity is advantageous as otherwise the modes could start to overlap in frequency and would not be able to be tracked. This opens possibility for multi-parameter sensing as each mode would detect different parameter.

## 4. Conclusions

In conclusion we suggest a new approach to obtaining ultra-high-*Q* photonic crystal slab microcavities via air-hole infiltration in otherwise defect-free PCS. We demonstrate that these cavities support a large number of high-*Q* modes with a strong localization and relatively small modal volume—comparable indeed with the best results reported to date in other types of cavities. This type of high-*Q* cavity originates from an air-band which is a key advantage for sensing applications. Even though we considered mostly infiltration by water, in principle any liquid can be used.

## Figures and Tables

**Table 1.** Quality factor, frequency, wavelength, wavelength spacing between neighbouring high-*Q* modes and modal volume for a periodicity of *a* = 480 nm. S stands for symmetric and A stands for anti-symmetric in the (*x,y,z*) directions. The index *i* represents group of modes, with *i = 0* represents the group of modes close to the shifted band edge and *i* = 3 the group closest to the original band edge.

	***Q(×10^6^)***	**Symmetry**	***ω=a/λ***	***λ*(nm)**	***Δ λ*(nm)**	***V (λ/n)^3^***
1	1.04	SSS_0_	0.31148	1541.0	0	3.63
2	1.08	ASA_0_	0.31176	1539.6	1.4	2.76
3	1.34	ASS_0_	0.31239	1536.5	3.1	4.89
4	1.41	SSA_0_	0.31284	1534.3	2.2	2.06
5	2.14	SSS_1_	0.31378	1529.7	4.6	2.11
6	2.05	ASA_1_	0.31400	1528.7	1.0	1.79
7	2.07	ASS_1_	0.31571	1520.4	8.3	2.83
8	2.73	SSA_1_	0.31616	1518.2	2.2	3.69
9	1.22	ASA_2_	0.31763	1511.2	7.0	1.43
10	0.49	SSS_2_	0.31797	1509.6	2.6	1.44
11	0.55	SSA_2_	0.31959	1501.9	7.7	3.34
12	1.49	SSA_2_	0.32032	1498.5	3.4	1.68
13	0.26	SSS_3_	0.32261	1487.9	10.6	1.68
14	1.01	ASA_3_	0.32268	1487.5	0.4	2.08
15	0.21	ASS_3_	0.32374	1482.7	4.8	1.17
16	0.79	SSA_3_	0.32412	1480.9	1.8	2.49
